# Identification and Characterization of Sepsis Phenotypes in an Indian Cohort

**DOI:** 10.1155/ccrp/9921379

**Published:** 2026-06-05

**Authors:** Fabia Edathadathil, Georg Gutjahr, Dipu Sathyapalan, Sanjeev Singh

**Affiliations:** ^1^ Department of Health Science Research, Amrita Institute of Medical Sciences and Research Centre, Amrita Vishwa Vidyapeetham, Kochi, Kerala, India, amrita.edu; ^2^ Department of Internal Medicine and Division of Infectious Diseases, Amrita Institute of Medical Sciences and Research Centre, Amrita Vishwa Vidyapeetham, Kochi, Kerala, India, amrita.edu; ^3^ Amrita Institute of Medical Sciences and Research Centre, Amrita Vishwa Vidyapeetham, Mata Amritanandamayi Marg RPS City Sector 88, Faridabad, Haryana, 121002, India, amrita.edu

## Abstract

**Background:**

Sepsis has a heterogenous clinical presentation with distinct regional epidemiological profiles. The study aims to identify clinical phenotypes from an Indian sepsis cohort.

**Methods:**

The cohort included all adult patients admitted through the emergency department with a presumed diagnosis of sepsis following Surviving Sepsis Guidelines criteria. The unsupervised k‐means clustering method was used, and the identified Indian clusters were compared using distance matching to phenotypic clusters in Seymour et al., which had previously identified four reproducible sepsis phenotypes in large Western ICU cohorts. The association of focus of infection and outcomes among the clusters were assessed.

**Results:**

Of the 1009 patients, four clusters were identified. Age (*p* = 0.024) and gender (*p* = 0.04) significantly differed among the clusters. The Indian clusters that exhibited close association to the alpha, beta, gamma, and delta phenotypes reported in Seymour et al. were termed i‐Alpha (254, 25%), i‐Beta (141, 14%), i‐Gamma (389, 39%), and i‐Delta (225, 22.3%) respectively. The clusters demonstrated significant variations in clinical profiles and outcomes. Septic shock and in‐hospital mortality were highest among i‐Alpha at 19% (48/254) and i‐Beta at 34% (48/141) (*p* < 0.001), respectively. Prevalence of pneumonia (*p* = 0.018), SSI (*p* = 0.011), and bacteremia (*p* = 0.004) significantly differed among the clusters. *Pseudomonas* (*p* = 0.008) and *Staphylococcus aureus* (*p* = 0.02) were also observed to be significantly different. i‐Gamma was characterized by a lower mortality rate and a high prevalence of soft tissue infections.

**Conclusion:**

Understanding these unique phenotypes can guide personalized treatment and improve sepsis outcomes in resource‐limited settings, highlighting the importance of localized research in sepsis management.

## 1. Introduction

Sepsis has been a complex syndrome of dysregulated host immune response that can lead to organ dysfunction and associated with high mortality and morbidity [1]. The heterogenous presentation of sepsis syndrome in terms of its variability in signs and symptoms, etiology and focus of infection, clinical outcomes, and response to treatment have led to numerous research efforts in identifying clinical phenotypes and inherent patient clusters [2]. Albeit tremendous research on biomarkers to predict onset of sepsis or severity, identifying distinct clinical profiles or heterogenous clusters within sepsis patients will potentially help in better prognosticating patients with poor outcomes or aid in tailoring treatment plans. This would also pave way for informing and strategizing personalized treatment and enhance clinical trial designs [3, 4].

The presentation of sepsis and clinical characteristics of sepsis vary in countries due to regional epidemiological profiles [5, 6]. The burden and clinical outcomes such as mortality rates for sepsis exhibited significantly varied estimates between high income countries (HICs) and lower middle income countries (LMICs) of different economical strata in research studies owing to regional disparities. LMICs are markedly characterized by in limited healthcare resources, lack of standardized clinical practices, higher sepsis prevalence rates, and higher proportion of multidrug‐resistant (MDR) pathogens that also differ at regional levels [4, 7]. The mortality and morbidity due to sepsis have been reported to be higher in LMICs with infections being the primary cause of mortality when compared with countries of higher economic indices where sepsis deaths were majorly attributable to noncommunicable diseases [1].

In HICs, numerous research studies on clustering sepsis patients have utilized a vast and diverse repertoire of variables including clinical characteristics (hemodynamic traits and multiorgan dysfunction), immunomodulatory parameters (cytokines and inflammatory markers), and genomic data (transcriptomic analysis) [8–11]. Data sources for clustering ranged from openly available datasets related to sepsis such as clinical registries, Electronic Health Record (EHR) databases of hospitals, and clinical trial patient data [2]. Different modeling methodologies such as unsupervised consensus clustering, latent class analysis, hierarchical clustering, and causal models were used for clustering analysis. Seymour et al. conducted a landmark analysis of large ICU datasets to identify four distinct sepsis phenotypes in the United States using unsupervised clustering. These phenotypes—Alpha, Beta, Gamma, and Delta—were shown to differ significantly in clinical characteristics, inflammatory profiles, and outcomes, demonstrating the biological and prognostic heterogeneity of sepsis [12]. These phenotypes demonstrated that patients meeting the same diagnostic criteria for sepsis may represent biologically and clinically distinct subgroups with potential implications for prognosis and treatment response. Whether these phenotypes are generalizable to low‐ and middle‐income country settings, such as India, remains largely unexplored. Another study used gene expression profiles from the blood samples of sepsis patients for identifying sepsis endotypes using unsupervised consensus clustering [13]. A few studies evaluated the developed models across external validation cohorts, but uses of prospective cohorts were limited [12–15]. Studies reported significant differences across the clusters in their profiles of inflammatory responses, infectious site and presentations, coagulopathy, severity of sepsis, pathophysiological progress, response to treatment, and mortality. The identification of hyperinflammatory clusters and temperature trajectory–based clusters differing on outcomes have led to potential clinically interpretable observations that would be helpful in tailoring therapeutic management.

Most prior studies on sepsis phenotyping have relied on publicly available critical care datasets from HICs, with limited representation from low‐ and middle‐income settings [12]. The dearth of sepsis data from countries of low sociodemographic indices has been highlighted in sepsis policy statements and guidelines [16, 17]. Data from India on sepsis incidence, outcomes, and phenotypic heterogeneity remain sparse, and it is unclear whether phenotypes described in high‐income populations are applicable to Indian cohorts with distinct epidemiological profiles. None of the models have been validated in LMICs. No comprehensive studies have explored and characterized sepsis phenotypes exclusively using Indian data.

Given the possible contribution of regional sepsis profiles to clinical outcomes and lack of investigation into sepsis phenotypic clusters among Indian patients, our study aims to identify and estimate the prevalence of clinical phenotypes from retrospective data of sepsis patients from a South Indian registry. We have attempted to compare the well‐described phenotypes reported by Seymour et al. to the phenotypes observed in our study.

## 2. Materials and Methods

### 2.1. Study Design and Setting

The study was conducted as a secondary analysis of a previously published Indian cohort of sepsis patients—Amrita Sepsis Cohort for Outcome and Phenotype Evaluation (A‐SCOPE) [18]. The single‐center study was carried out at a 1320‐bedded university‐affiliated hospital, and ethical approval for the study was obtained from the Institutional Ethics Committee of the hospital.

### 2.2. Data Collection

Data of sepsis patients were obtained from the in‐hospital electronic sepsis registry [18]. The registry contains the clinical information and outcomes of all adult patients admitted through the emergency department with a presumed diagnosis of sepsis, as per Sepsis‐2 criteria following the Surviving Sepsis Guidelines (SSG) [19–21]. Variables used in the clustering algorithm included demographics, clinical investigations, laboratory parameters, and microbiological details.

### 2.3. Data Preparation

For variables with missing data, we assumed the data were “missing at random.” For a statistically valid imputation of missing values, we performed multiple imputations using the multivariate imputation by chained equations (MICE) method that fills missing values based on observed relations with other variables in the study cohort [22]. The next preprocessing step was performing one‐hot encoding for unordered categorical variables for accurate data characterization and relationships. One‐hot encoding converts text‐based categories into numerical figures as part of the requisite for machine‐learning model.

### 2.4. Clustering Analysis

We first explored whether the data contained natural groups using the Ordering Points to Identify the Clustering Structure (OPTICS) assessment [23]. This assessment helps to select the optimal clustering algorithm based on a reachability plot that aids in comprehensive visualization of the underlying data structure and supports in identifying appropriate clustering approaches. Based on the reachability plot, k‐means clustering was selected as the unsupervised clustering method. Unsupervised clustering methods identify patterns in data without predefining outcomes or categories by grouping data into clusters. In order to select the optimal number of clusters, we used the Hubert statistics which provides a graphical method to evaluate how well patients are separated into distinct groups based on the number of clusters [24]. The optimal number of clusters was validated using the Elbow method [25]. For k‐means clustering, the Hartigan–Wong algorithm was applied with 20 random starting points to ensure a stable identification of cluster centers [26].

### 2.5. Cluster Validation

Cluster stability was assessed as part of cluster validation using a bootstrap resampling approach to evaluate the robustness of the identified phenotypes. We generated 1000 bootstrap samples from the original dataset and repeated the clustering procedure for each sample. The consistency of patient assignment across the original and resampled datasets was measured using the Jaccard index, with higher values indicating greater stability [27, 28].

### 2.6. Comparison of the Indian Clusters with Seymour et al.

The sepsis clusters identified in the Indian cohort were compared with the clusters identified by a study in the United States by Seymour et al. The four clusters identified in the study were Alpha, Beta, Gamma, and Delta phenotypes. Cluster matching between Indian clusters and phenotypes reported by Seymour et al. was performed using a distance‐based approach (*Optmatch* package, R), which pairs clusters based on similarity across clinical and laboratory variables. Mahalanobis distance was used to account for correlations between variables, enabling optimal matching of each Indian cluster to a corresponding Seymour phenotype.

### 2.7. Statistical Analysis

Descriptive statistics was used to summarize the major prevalence rates and key indicators of the clusters in terms of frequencies, proportions, and dispersion indices. Continuous and categorical variables were evaluated between the clusters using ANOVA, Kruskal–Wallis tests, and chi‐square tests based on their distributions. Both the variables that were used in the clustering as well as additional variables such as clinical outcomes were compared across clusters. A *p* value of < 0.05 was considered to be statistically significant. All statistical analyses were performed using the R statistical software Version 1.3.1093.

## 3. Results

The retrospective analysis included 1009 patients. The most common age group was 61–80 years (41%) and the majority of patients were male (70%), as recorded in the in‐house e‐sepsis registry over a study period of three years. The baseline characteristics of the study cohort are furnished in Supporting Table S1 as previously reported [18].

### 3.1. Cluster Analysis and Baseline Characteristics

**FIGURE 2:**
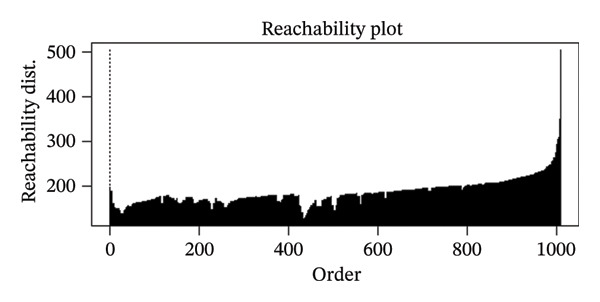
Reachability plot to assess the clustering structure.

Hubert statistics and the Elbow method were used to select the number of clusters in the Indian cohort (Figures 1(a) and 1(b)). Four clusters were observed in the Indian cohort. The selection of the clustering algorithm was based on the reachability plot generated by the OPTICS method (Figure 2). The four Indian clusters were matched to the Alpha, Beta, Gamma, and Delta phenotypes derived by Seymour et al. using distance‐based matching. The four Indian clusters were labeled i‐Alpha (Indian Alpha), i‐Beta, i‐Gamma, and i‐Delta. Among the matched pairs, the closest correspondence was observed between the Delta and i‐Delta (Mahalanobis distance: 2.87), followed by Beta and i‐Beta (4.31), Alpha and i‐Alpha (5.51), and Gamma and i‐Gamma (6.34). The comparisons of major baseline variables between the paired phenotypes are given in Table 1.

FIGURE 1(a) Hubert statistics to determine the ideal number of clusters. (b) Elbow method to determine the ideal number of clusters.

**Figure:**
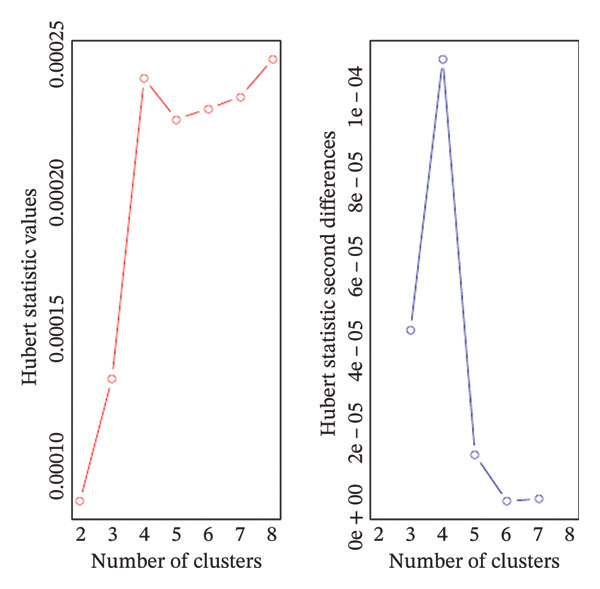
(a)

**Figure:**
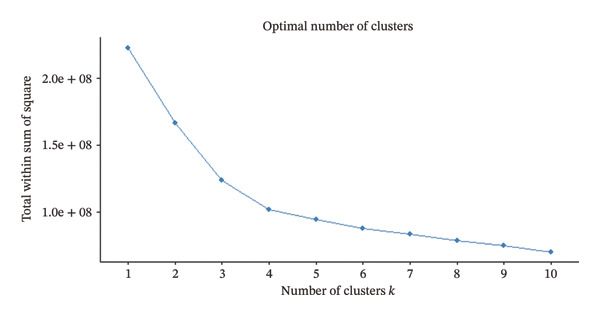
(b)

**TABLE 1:** Comparison of clinical characteristics of corresponding sepsis clusters of Indian cohort with Seymour et al.

Characteristics	Alpha	i‐Alpha	Beta	i‐Beta	Gamma	i‐Gamma	Delta	i‐Delta
Age	60 (18)	56 (15)	71 (15)	60 (15)	65 (16)	58 (16)	63 (17)	56 (17)
Males	3372 (51)	188 (74)	2624 (48)	106 (75)	2559 (48)	254 (65)	1467 (55)	155 (69)
Females	3253 (49)	66 (26)	2888 (52)	35 (25)	2826 (52)	135 (35)	1200 (45)	70 (31)
SOFA score	3.0 (1.4)	7.5 (3.0)	3.5 (1.7)	7.3 (3.4)	4.0 (2.3)	5.8 (3.1)	6.6 (3.7)	6.3 (3.1)
CRP (mg/dL)	2 (0.4–6)	8 (9.2)	5 (2–12)	20.3 (11.7)	16 (9–32)	9.1 (8.5)	13 (4–30)	9.0 (9.5)
White blood cell count, median (IQR), × 10^9^/L	9 (6–12)	12.2 (11.9)	9 (7–13)	13.9 (8.9)	11 (7–16)	14.59 (11.1)	12 (8–18)	12.5 (7.9)
Temperature, mean (SD), °C	37.1 (0.9)	37.4 (0.7)	36.7 (0.8)	37.4 (0.7)	37.3 (1.0)	37.4 (0.7)	36.7 (1.3)	37.3 (0.7)
Respiratory rate, mean (SD), breaths/min	20 (4)	23 (6)	20 (4)	25 (7)	25 (7)	23 (6)	25 (8)	24 (6)
Heart rate, mean (SD), beats/min	94 (19)	98 (18)	84 (16)	108 (22)	109 (21)	102 (18)	108 (24)	104 (21)
Serum lactate, median (IQR), mmol/L	1.3 (1.0–1.9)	2.4 (2.0)	1.2 (0.9–1.8)	2.5 (1.8)	1.8 (1.2–2.7)	2.1 (1.8)	3.3 (2.0–5.7)	2.2 (2.0)
Bicarbonate, mean (SD), mEq/L	27 (4)	20 (7)	25 (5)	19 (5)	25 (5)	20 (6)	20 (5)	19 (7)
Systolic blood pressure, median (IQR), mm Hg	118 (104–134)	121 (106–139)	120 (103–138)	121 (100–144)	99 (83–113)	133 (115–154)	91 (77–109)	129 (110–150)
Platelets, median (IQR), × 10^9^/L	179 (128–246)	75 (49–124)	200 (143–263)	146 (77–181)	195 (131–269)	301 (222–400.1)	164 (104–241)	200 (108–286)
In‐hospital mortality, no. (%)	126 (2)	80 (32)	286 (5)	48 (34)	818 (15)	61 (16)	852 (32)	60 (27)

i‐Alpha patients were characterized by a high incidence of septic shock and bacteremia, with comparatively lower platelet counts and worse clinical outcomes than the Alpha phenotype described by Seymour et al. i‐Beta represented the smallest but highest‐risk group, consisting of older patients with a high prevalence of pneumonia, hypoxemia, and bacteremia. This phenotype exhibited the highest in‐hospital mortality. i‐Gamma, the most prevalent phenotype, was associated with soft tissue infections, higher white blood cell and platelet counts, preserved hemodynamics, and the lowest mortality, suggesting a relatively favorable clinical course. i‐Delta was marked by a high prevalence of *Pseudomonas* infections and had a similar profile of mortality rates and vital signs on admission to the Delta phenotype reported by Seymour et al.

Bootstrap validation revealed stable cluster derivations with cluster‐wise Jaccard bootstrap mean at 0.70, 0.77, 0.50, and 0.90 for i‐Alpha, i‐Beta, i‐Gamma, and i‐Delta, respectively.

### 3.2. Baseline Characteristics

Among the four clusters identified, i‐Gamma was the most prevalent (39%), followed by i‐Alpha (25%), i‐Delta (22.3%), and i‐Beta (14%). The major baseline characteristics of the study cohort are depicted in Table 2. Clinical values and distribution of vital signs based on the clinical cutoff ranges are provided in Supporting Table S2. i‐Beta harbored the oldest patients (mean age: 59.82 ± 15.3 years) (*p* = 0.024) and exhibited the highest male predominance (75%), while i‐Gamma had the lowest proportion of male patients (65%).

**TABLE 2:** Distribution of baseline clinical characteristics and microbiological variables used for clustering among the derived phenotypes.

Parameters	i‐Alpha (254, 25.2%)	i‐Beta (141, 14%)	i‐Gamma (389, 39%)	i‐Delta (225, 22.3%)	*p*
*Seymour* et al. *phenotype match*	Alpha	Beta	Gamma	Delta	
Age	55.31 ± 14.5	59.82 ± 15.3	58.23 ± 16.8	56.21 ± 16.5	0.02
Age strata					0.002
18–40	40 (16%)	18 (13%)	63 (16%)	41 (18%)	
41–60	117 (46%)	41 (29%)	131 (34%)	82 (36%)	
61–80	86 (34%)	78 (55%)	167 (43%)	89 (40%)	
80 and above	11 (4.3%)	4 (2.8%)	28 (7.2%)	13 (5.8%)	
Gender					
Females	66 (26)	35 (25)	135 (35)	70 (31)	0.04
Males	188 (74)	106 (75)	254 (65)	155 (68)	
Heart rate	98.17 ± 18.4	108.25 ± 22.5	101.97 ± 18.6	103.65 ± 21.02	< 0.001
Pulse pressure	52.2 ± 18.68	49.93 ± 18.4	59.09 ± 53.59	55.3 ± 21.7	0.03
SBP	123.52 ± 25.94	122.69 ± 29.04	136.98 ± 57.02	130.61 ± 27.1	< 0.001
DBP	71.40 ± 18.06	72.76 ± 19.19	77.89 ± 17.2	75.13 ± 16.17	< 0.001
WBC	11.85 ± 10.43	13.98 ± 8.8	14.61 ± 11.18	12.47 ± 7.88	< 0.001
Platelet	89.42 ± 56.4	133.56 ± 72.51	318.57 ± 141.63	256.76 ± 140.7	< 0.001
NLR	11.11 ± 13.37	13.97 ± 11.95	9.37 ± 11.44	13.86 ± 36.05	0.02
MLR	1.16 ± 1.97	0.97 ± 1.2	0.63 ± 0.54	0.73 ± 0.82	< 0.001
PLR	119.29 ± 157.40	204.24 ± 231.80	250.12 ± 222.47	282.08 ± 595.54	< 0.001
Charlson Comorbidity Score	4.00 (3.00, 5.00)	4.00 (2.00, 5.00)	4.00 (2.00, 6.00)	4.00 (2.00, 6.00)	0.9
Score 0–3	107 (42)	65 (46)	171 (44)	104 (46)	0.7
4 and greater	144 (57)	76 (54)	216 (56)	120 (53)	
pH	7.42 ± 0.09	7.36 ± 0.66	7.69 ± 4.98	7.31 ± 0.72	0.48
HCO_3_	20.14 ± 7.07	18.85 ± 5.51	20.84 ± 5.94	19.32 ± 6.87	0.01
Lactate	2.44 ± 2.06	2.45 ± 1.77	2.1 ± 1.86	2.23 ± 2.05	0.14
SOFA	7.48 ± 3.03	7.26 ± 3.42	5.84 ± 3.0	6.31 ± 3.1	< 0.001
Altered mental status	62 (16)	38 (10)	80 (21)	51 (13)	
Focus of infection					
UTI	95 (37)	46 (33)	139 (36)	84 (37)	0.78
Bacteremia	74 (29%)	41 (29%)	75 (19%)	43 (19%)	0.04
Pneumonia	42 (17%)	40 (28%)	101 (26%)	52 (23%)	0.02
Skin and soft infections	16 (6.3%)	16 (11%)	57 (15%)	23 (10%)	0.01
Distribution of organisms					
*Klebsiella*	61 (24)	28 (20)	78 (20.1)	44 (20)	0.57
*E. coli*	44 (17)	33 (23)	69 (18)	38 (17)	0.39
Acinetobacter	14 (6)	9 (6)	14 (4)	13 (6)	0.45
Candida non albicans	19 (7)	13 (9)	43 (11)	28 (12)	0.29
*Enterococcus*	28 (11)	17 (12)	38 (10)	20 (9)	0.74
*Staphylococcus aureus*	19 (7)	8 (6)	15 (4)	4 (2)	0.02
*Pseudomonas*	15 (6)	10 (7)	28 (7)	31 (14)	0.01
Proteus	3 (1)	2 (1)	6 (2)	3 (1)	0.98
Others	51 (20)	21 (15)	98 (25)	44 (20)	0.05
Overall Gram‐negative organisms					0.06
Negative	96 (38%)	67 (48%)	134 (34%)	86 (38%)	
Others	158 (62%)	74 (52%)	255 (66%)	139 (62%)	
Overall Gram‐positive organisms					0.12
Others	210 (83%)	120 (85%)	344 (88%)	200 (89%)	
Positive	44 (17%)	21 (15%)	45 (12%)	25 (11%)	
Overall fungal organisms					0.38
Fungal	18 (7.1%)	13 (9.2%)	40 (10%)	26 (12%)	
Others	236 (93%)	128 (91%)	349 (90%)	199 (88%)	
MDR distribution					
Total MDR	80 (32)	53 (37)	116 (30)	65 (29)	0.31
CRE	30 (12)	15 (11)	40 (10)	35 (16)	0.25
ESBL positive	28 (11)	21 (15)	38 (10)	18 (8)	0.19

Among the vital signs recorded on admission, a significant difference was noted in heart rates (*p* < 0.001) with the highest mean heart rate (108.25 ± 22.5 bpm) and the greatest prevalence of tachycardia (62%) noted in i‐Beta patients, while i‐Alpha had the lowest mean heart rate (98.17 ± 18.4 bpm). Blood pressure profiles also varied, with i‐Gamma exhibiting the highest mean systolic and diastolic pressures (136.98 ± 57.02/77.89 ± 17.2 mm Hg), whereas the severely ill i‐Beta and i‐Alpha patients showed lower values. Temperature on admission and altered mental status did not significantly differ between the clusters.

Hematological parameters differed significantly across clusters. i‐Alpha exhibited the lowest mean WBC (11.85 ± 10.43 × 10^9^/L) and platelet counts (89.42 ± 56.4 × 10^9^/L), while i‐Gamma had the highest counts of WBC (14.61 ± 11.18 × 10^9^/L) (*p* < 0.001) and platelet (318.57 ± 141.63 × 10^9^/L). Among inflammatory ratios, neutrophil to lymphocyte ratio (NLR) was significantly higher in i‐Beta (13.97 ± 11.95) and i‐Delta (13.86 ± 36.05). Monocyte to lymphocyte ratio (MLR) values were significantly higher in i‐Alpha (1.16 ± 1.97) and i‐Beta (0.97 ± 1.2), the clinically sicker cohorts.

Platelet to lymphocyte ratio (PLR) also exhibited significant difference among clusters, highest in i‐Delta (282.08 ± 595.54) and lowest in i‐Alpha (119.29 ± 157.40).

Prevalence of comorbidities, assessed by Charlson Comorbidity Index (CCI), did not exhibit a significant difference across clusters (*p* = 0.7) although higher CCI scores (≥ 4) were more common in i‐Alpha (57%). Among arterial blood gas (ABG) parameters, HCO_3_ (*p* = 0.006) and PaO_2_ (*p* < 0.001) significantly differed among clusters with lowest mean values observed in i‐Beta at 18.85 ± 5.51 and 69.24 ± 33.01, respectively. Baseline characteristics of the four clusters in terms of continuous and categorical variables are given in Figures 3(a) and 3(b), respectively.

FIGURE 3(a) Comparison of clinical parameters across the clusters. (b) Comparison of categorical and derived variables across the clusters.

**Figure:**
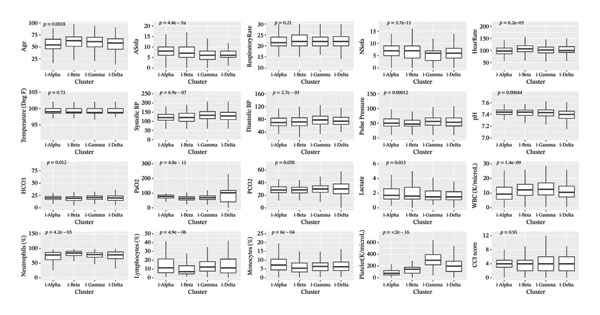
(a)

**Figure:**
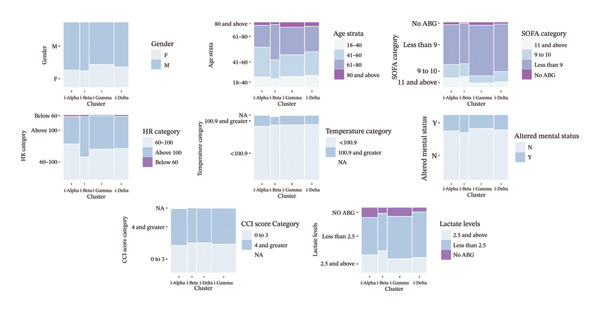
(b)

With respect to the focus of infection, bacteremia was observed to be significantly different among the clusters with highest prevalence in i‐Beta and i‐Alpha (29% each; *p* = 0.004), while i‐Beta also had the highest prevalence of pneumonia (28%; *p* = 0.018) (Figure 4(a)). Skin and soft tissue infections (SSIs) significantly differed among the clusters with highest prevalence in i‐Gamma (15%), followed by i‐Beta (11%; *p* = 0.011). Urinary tract infections were frequent in i‐Alpha and i‐Delta (37% each) but did not differ significantly across clusters.

FIGURE 4(a) Distribution of focus of infection among the derived clusters. (b) Distribution of pathogens among the derived clusters of the cohort.

**Figure:**
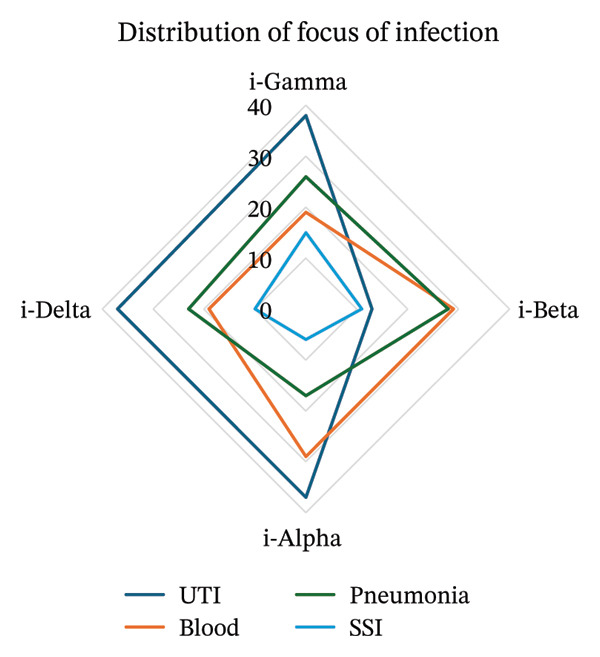
(a)

**Figure:**
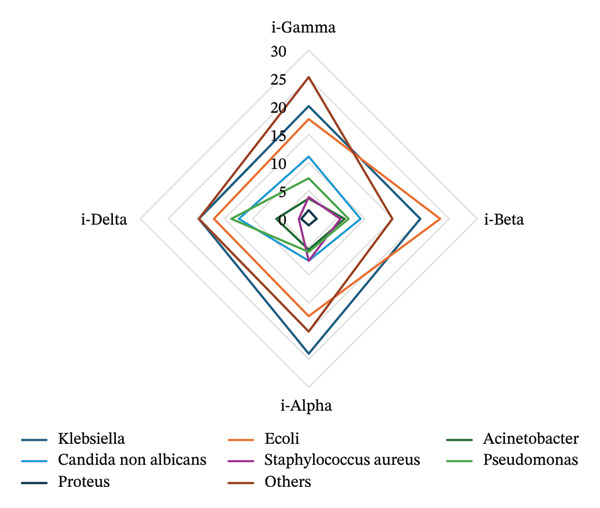
(b)

The distribution of predominant pathogens varied across clusters. The predominant pathogens such as *Klebsiella* and *E. coli* species did not significantly differ among the clusters, with the highest proportions observed in i‐Alpha (*Klebsiella* at 24%) and i‐Beta (*E. coli* at 23%) (Figure 4(b)). In contrast, *Pseudomonas* species differed significantly among the clusters, with the highest prevalence in i‐Delta at 13.7% (*p* = 0.008). *Staphylococcus aureus* species also varied significantly, occurring most frequently in i‐Alpha at 7.4% (*p* = 0.02). The overall distribution of Gram‐negative and Gram‐positive strains did not significantly differ between the clusters. i‐Beta had the highest proportion of patient with a reported microbiological culture positive for Gram‐negative infections at 48% (67). i‐Alpha had the highest proportion of Gram‐positive organisms at 17% (44). Fungal infections were uncommon and did not differ significantly overall although i‐Delta had the highest prevalence at 12%. MDR pathogens were similar among the clusters, with the highest proportion in i‐Beta at 37.6%, followed by i‐Alpha at 31.5%. The distribution of carbapenem resistance and ESBL positivity also revealed no significant difference among the clusters. CRE strains were highly prevalent among i‐Delta (15.6%, 35) and i‐Alpha (11.8%, 30). In case of patients with UTI, CRE significantly differed across the clusters with i‐Gamma recording the highest proportion at 37% (*p* = 0.043).

### 3.3. Association With Clinical Outcomes

In‐hospital mortality differed significantly across phenotypes (*p* < 0.001), highest in i‐Beta (34%) followed by i‐Alpha (32%) and lowest in i‐Gamma (16%) (*p* < 0.001). Table 3 depicts in‐hospital mortality and sepsis severity. Septic shock also exhibited a significant difference among the clusters with highest proportion in i‐Alpha (19%), followed by i‐Beta (18%) (*p* < 0.001).

**TABLE 3:** Distribution of outcomes among derived clusters.

Outcomes	i‐Alpha (254, 25.2%)	i‐Beta (141, 14%)	i‐Gamma (389, 39%)	i‐Delta (225, 22.3%)	*p*
Septic shock	48 (19)	26 (18)	31 (8)	31 (14)	< 0.001
2012 SSC guideline sepsis severity classification					< 0.001
Sepsis	68 (27)	37 (26)	211 (54)	96 (42)	
Severe sepsis	138 (54)	78 (55)	147 (38)	98 (43)	
Shock	48 (19)	26 (18)	31 (8)	31 (14)	
In‐hospital mortality	80 (31)	48 (34)	61 (16)	60 (27)	< 0.001

## 4. Discussion

The study identified four distinct clinical phenotypes of sepsis within an Indian cohort that exhibited significant differences in demographics, infection patterns, and clinical outcomes. We compared the Indian clusters to the phenotypes reported by Seymour et al. and characterized the similarities and differences observed [12]. i‐Alpha was associated with a higher incidence of septic shock, i‐Beta with the highest in‐hospital mortality and frequent pneumonia and bacteremia, i‐Gamma with predominant skin and soft tissue infections and more favorable outcomes, and i‐Delta with *Pseudomonas* infection. These findings underscore the heterogeneity of sepsis in the Indian context and the need for targeted management strategies.

The Indian clusters agreed with many characteristics with those reported by Seymour et al., despite some differences in severity and outcomes. Even the i‐Gamma and gamma cluster, despite the largest Mahalanobis distance, had similar mortality rates (16% vs. 15%). Beta phenotypes were older and exhibited higher blood pressures suggestive of a hyperdynamic state though mortality and organ dysfunction differed. Though i‐Alpha resembled Alpha on vital signs (HR, temperature, SBP, and RR), i‐Alpha suffered much worse clinical outcomes compared with the Alpha phenotype and the Delta pair resembled on vital signs, lab measurements, and clinical outcomes. However, a subsequent analysis linked the Delta phenotype described by Seymour et al. to the hyperinflammatory phenotype reported by DeMerle et al., whereas this association could not be clearly established for i‐Delta as it did not exhibit high CRP levels despite having the highest NLR values among clusters [29].

In addition to the comparison with Seymour et al., the Indian clusters were also compared with other reported studies. Regarding focus of infection, i‐Beta with highest prevalence of pneumonia, lowest PaO_2_ levels, and an increased proportion of septic shock (18%) showed similarity to “shock with hypoxemia” cluster reported by Knox et al. [3]. In the same study, i‐Alpha resembled “shock with elevated creatinine” cluster was observed to be similar to i‐Alpha in terms of higher prevalence of UTI and septic shock. i‐Beta also resembled the M1 phenotype reported by Aldewereld et al. with highest prevalence of pneumonia and high lactate levels. i‐Gamma mirrored lower‐risk phenotypes described by Aldewereld and Kudo et al. characterized by a higher prevalence of skin and soft tissue infections and lower SOFA scores [30, 31].

Epidemiological data from India exhibit a distinct pathogenic profile, in terms of Gram staining status, pathogenic organisms, and multidrug resistance patterns typical of LMICs [4, 32]. In terms of pathogenic profile, i‐Beta was similar to host pathogen *γ*‐type reported by Zhao et al. [33].

Interestingly, *Staphylococcus aureus* was significantly higher in i‐Alpha that harbored highest proportion of septic patients and patients with bacteremia, even though i‐Gamma had the highest prevalence of SSI. In India, Staphylococci are involved in 20.7% of superficial infections and 19% of bacteremia as per Indian Council of Medical Research surveillance data [34]. On contrary to *Staphylococcus*, *Pseudomonas* was significantly higher in i‐Delta and low in i‐Alpha that could potentially indicate their antagonistic interactions in our cohort [35].

The observed differences between clusters may be influenced by a combination of host factors and epidemiological and environmental factors. The high prevalence of *Pseudomonas* in i‐Delta and *Staphylococcus aureus* in i‐Alpha could be attributed to regional microbial resistance patterns and healthcare‐associated infections. The i‐Beta cluster, which shows a high incidence of respiratory infections and severe outcomes, could be linked to vulnerabilities in elderly patients and the prevalence of MDR organisms, exacerbating the clinical severity. Variations in immune responses, as indicated by differing levels of inflammatory markers across clusters, may also play a role in determining clinical outcomes. Further research into genetic and biomarker profiles of these clusters could discern the pathophysiological mechanisms underlying these differences.

The identification of distinct sepsis phenotypes within the Indian cohort has several clinical implications. First, these clusters allow early risk stratification and more targeted management, such as prioritizing intensive monitoring for high‐mortality i‐Beta patients, focusing on infection source control and antibiotics in i‐Gamma with skin and soft tissue infections, aggressive hemodynamic support in i‐Alpha, and early broad‐spectrum therapy in i‐Delta. Overall, phenotype‐based stratification may enable more personalized and effective sepsis care than uniform treatment approaches.

This study has limitations, including reliance on admission‐time variables without capturing dynamic disease progression, lack of genotype or endotype analyses that were beyond the scope of the present study, and a single‐center design that may limit generalizability. Future studies need to explore the potential of the identified phenotype for informing clinical decision. Studies should prospectively validate these phenotypes in multicenter cohorts and assess their clinical utility across Indian and other LMIC settings.

## 5. Conclusion

The study identified four distinct sepsis phenotypes in an Indian cohort. These clusters showed strong similarities with phenotypes identified in HIC setting. However, clusters also exhibited unique regional differences underscoring the impact of local epidemiological factors. This is one of the pioneer studies in demonstrating inherent subgroups among sepsis patients within an Indian cohort data that significantly differed in outcomes and epidemiology.

## Funding

The study did not receive any funding from financial organizations or funding agencies.

## Conflicts of Interest

The authors declare no conflicts of interest.

## Supporting Information

Additional supporting information can be found online in the Supporting Information section.

## Supporting information


**Supporting Information 1** Supporting Table S1: Baseline demographics and disease characteristics.

**Supporting Information 2** Supporting Table S2: Clinical characteristics and categorized vital signs.

## Data Availability

Data used to support the findings of this study are available from the corresponding author upon reasonable request.
