# Supplementary material for: Identification and Characterization of Sepsis Phenotypes in an Indian Cohort
Source: Crit Care Res Pract. 2026 Jun 5;2026:9921379. doi: 10.1155/ccrp/9921379 (PMC13238234; doi:10.1155/ccrp/9921379)
Supplement: Supplementary file 1 — Supporting Information 1 Supporting Table S1: Baseline demographics and disease characteristics. [file CCRP-2026-9921379-s001.docx]

**Supplementary Table S1: Baseline demographics and disease characteristics.**

| **Characteristics** | **N (%)** |
| --- | --- |
| Age | |
| Mean | 57.37 ± 16.05 |
| 18–40 | 162 (16) |
| 41–60 | 371 (37) |
| 61–80 | 420 (41) |
| 80 and above | 56 (6) |
| Gender | |
| Females | 306 (30%) |
| Males | 703 (70%) |
| Outcomes | |
| Alive | 760 (75) |
| Expired | 249 (25) |
| Severity of sepsis | |
| Sepsis | 412 (41) |
| Severe sepsis | 461 (46) |
| Sepsis with shock | 136 (13) |
| CCI score | |
| Mean ± SD | 3.8 ± 2.4 |
| 0 to 2 | 285 (28) |
| 3 and above | 724 (72) |
| ASOFA scores | |
| Mean ± SD | 6.56 ± 3.2 |
| Asofa 0 to 1 | 16 (1) |
| Asofa 2 to 7 | 606 (60) |
| Asofa 8 to 11 | 303 (30) |
| Asofa above 11 | 54 (5) |
| No ABG | 30 (3) |
| Lactate levels | |
| 2.5 and above | 252 (24.9) |
| Less than 2.5 | 641 (63.5) |
| Heart rate | |
| Below 60 | 15 (1.5) |
| 60–100 | 488 (48) |
| Above 100 | 506 (50) |
| Temperature | |
| <100.9 | 862 (85) |
| 100.9 and greater | 143 (14) |
| Surviving Sepsis Campaign bundle compliance | |
| 3 h bundle compliance | 371 (37%) |
| 6 h bundle compliance | 128/260 (49%) |
| Focus of infection | |
| UTI | 364 (36) |
| Bacteremia | 233 (23) |
| Pneumonia | 235 (23) |
| SSI | 112 (11) |
| Culture positivity | 572 (57%) |
| Type of organism | |
| Gram-negative | 383 (67) |
| Gram-positive | 135 (24) |
| Fungal | 97 (9.6) |
| Pathogens isolated | |
| Klebsiella | 186 (29) |
| Ecoli | 182 (28) |
| Enterococcus | 99 (15) |
| Candida non albicans | 97 (15) |
| Pseudomonas | 58 (9) |
| Candida albicans | 51 (8) |
| Staphylococcus aureus | 46 (7) |
| Acinetobacter baumannii | 41 (6) |
| Streptococcus | 23 (4) |
| Proteus | 11 (2) |
| Multidrug resistance | 286 (28%) |
