# Supplementary material for: Identification and Characterization of Sepsis Phenotypes in an Indian Cohort
Source: Crit Care Res Pract. 2026 Jun 5;2026:9921379. doi: 10.1155/ccrp/9921379 (PMC13238234; doi:10.1155/ccrp/9921379)
Supplement: Supplementary file 2 — Supporting Information 2 Supporting Table S2: Clinical characteristics and categorized vital signs. [file CCRP-2026-9921379-s002.docx]

Supplemental Table S2: Clinical characteristics and categorized vital signs

| **Parameters** | **i-Alpha (254, 25.2%)** | **i-Beta (141, 14%)** | **i-Gamma (N=389, 39%)** | **i-Delta (225, 22.3%)** | **p** |
| --- | --- | --- | --- | --- | --- |
| *Seymour et al phenotype match* | **alpha** | **beta** | **gamma** | **delta** |  |
| PaO2 | 76.02± 14.2 | 69.24± 33.01 | 71.14± 30.58 | 95.94± 62.55 | <0.001 |
| PCO2 | 30.35± 16.86 | 29.72± 11.47 | 32.57± 17.83 | 33.34± 15.72 | 0.09 |
| Heart Rate category |  |  |  |  |  |
| Heart Rate below 60 bpm | 5 (2) | 3 (21) | 3 (0.7) | 4 (2) |  |
| Heart Rate between 60 - 100 bpm | 142 (56) | 50 (35) | 186 (48) | 110 (49) |  |
| Heart Rate above 100 bpm | 107 (42) | 88 (62) | 200 (51) | 111 (49) |  |
| Temperature category |  |  |  |  | 0.63 |
| Temperature category below 100.9 | 212 (83) | 121 (85) | 334 (85) | 195 (87) |  |
| Temperature at 100.9 and greater | 42 (17) | 17 (12) | 54 (14) | 30 (13) |  |
| Lactate Categorization |  |  |  |  | 0 |
| 2.5 and above | 69 (27%) | 48 (34%) | 83 (21%) | 52 (23%) | 0.01 |
| Less than 2.5 | 147 (58%) | 81 (57%) | 254 (65%) | 159 (71%) |  |
| NO ABG | 38 (15%) | 12 (8.5%) | 52 (13%) | 14 (6.2%) | - |
| SBP Greater than 120 |  |  |  |  | <0.001 |
| N | 114 (45%) | 65 (46%) | 118 (30%) | 84 (37%) |  |
| Y | 140 (55%) | 76 (54%) | 271 (70%) | 141 (63%) |  |
| DBP Greater than 80 |  |  |  |  | <0.001 |
| N | 174 (69%) | 87 (62%) | 202 (52%) | 135 (60%) |  |
| Y | 80 (31%) | 54 (38%) | 187 (48%) | 90 (40%) |  |
| RR Greater than 20 |  |  |  |  | 0.42 |
| N | 28 (11%) | 17 (12%) | 32 (8.2%) | 26 (12%) |  |
| Y | 226 (89%) | 124 (88%) | 357 (92%) | 199 (88%) |  |
| Pulse pressure Greater than 40 |  |  |  |  |  |
| N | 52 (20%) | 35 (25%) | 61 (16%) | 45 (20%) | 0.09 |
| Y | 202 (80%) | 106 (75%) | 328 (84%) | 180 (80%) |  |
